# Acute compartment syndrome of the forearm as a rare complication of toxic epidermal necrolysis: a case report

**DOI:** 10.1186/1752-1947-6-84

**Published:** 2012-03-20

**Authors:** Tamer Kamal, Sherif Elnikety, Heba Mashaly, James Casha

**Affiliations:** 1 Queen Elizabeth The Queen Mother Hospital, St Peters Road, Margate CT9 4AN, UK; 2 Kasr El Eini Hospital, Cairo University Hospital, Cairo, Egypt

## Abstract

**Introduction:**

Toxic epidermal necrolysis lies within the spectrum of severe cutaneous adverse reactions induced by drugs, affecting skin and mucous membranes. Toxic epidermal necrolysis is considered a medical emergency as it is considered to be potentially fatal and carries a high mortality rate. To the best of our knowledge the association of toxic epidermal necrolysis and compartment syndrome has been rarely mentioned in the literature. In this case we treated the compartment syndrome promptly despite the poor general condition and skin status of our patient. Despite the poor skin condition, wound healing was uneventful with no complications.

**Case presentation:**

A 62-year-old Caucasian man with a generalized macular-vesicular rash involving 90% of his body surface area and mucous membranes, as well as impaired renal and hepatic functions following ingestion of allopurinol for treatment of gout, was admitted to our hospital. Skin biopsies were taken and he was started on a steroid infusion. Within hours of admission, he developed acute compartment syndrome of the dominant forearm and hand.

**Conclusions:**

Despite its rare incidence, toxic epidermal necrolysis is a condition with a high incidence of complications and mortality. Patients with severe conditions affecting a large degree of the skin surface area should be treated as promptly and effectively as patients with burns, with close monitoring and the anticipation that rare musculoskeletal complications might arise. The association of compartment syndrome and toxic epidermal necrolysis might lead to a rapid deterioration and fatal systemic involvement and multiple organ failures.

## Introduction

Toxic epidermal necrolysis (TEN) lies within the spectrum of severe cutaneous adverse reactions (SCAR) induced by drugs, and affects the skin and mucous membranes [1,2]. TEN is considered a medical emergency as it may be potentially fatal and carries a high mortality rate. It is characterized by mucocutaneous tenderness and typically hemorrhagic erosions, erythema and more or less severe epidermal detachment presenting as blisters and areas of denuded skin [3]. In 1995, a case-control study assessed the medications that may be related to TEN [4]. According to a more recent case-control study, several drugs were confirmed to be at 'high risk' of inducing TEN, including: anti-infectives such as cotrimoxazole and sulfonamides, allopurinol, carbamazepine, phenytoin, phenobarbital and non-steroidal anti-inflammatory drugs (NSAIDs) of the oxicam type. Other drugs with 'significant but lower risk' included acetic acid NSAIDs, macrolides, quinolones, cephalosporins and tetracyclines [5]. Among these drugs, allopurinol is considered to be the most common cause of TEN in Europe [6,7]. Many previous studies have reported cases of allopurinol-induced TEN, and some have noted that the outcome of allopurinol-induced TEN is particularly lethal [8,9].

### Case presentation

**Figure 1:**
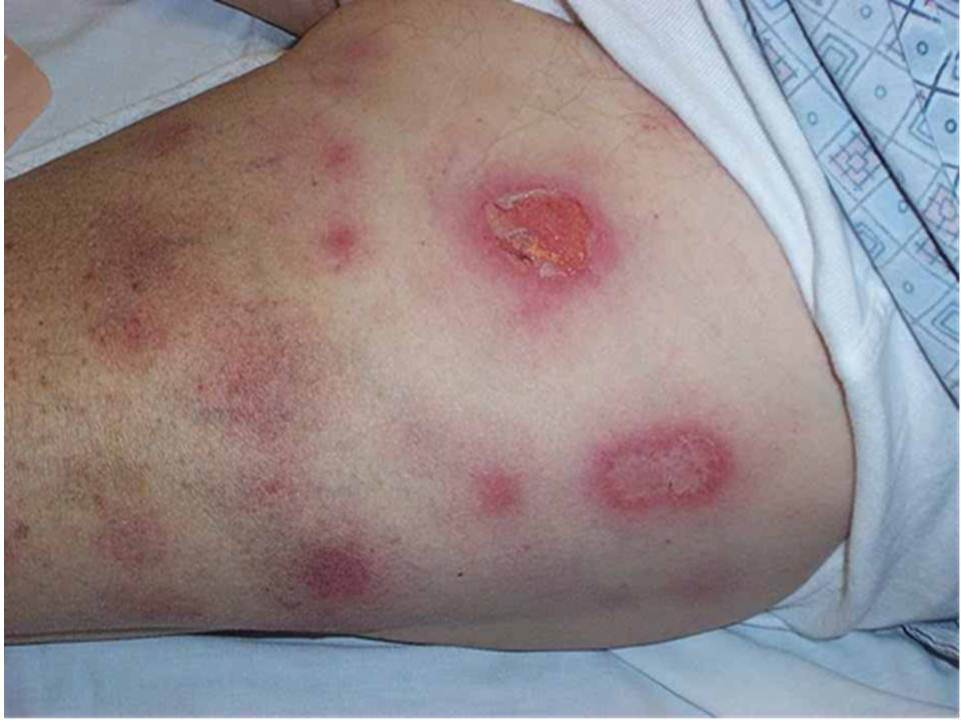
**Macropapular and vesicular drug eruptions**.

**Figure 2:**
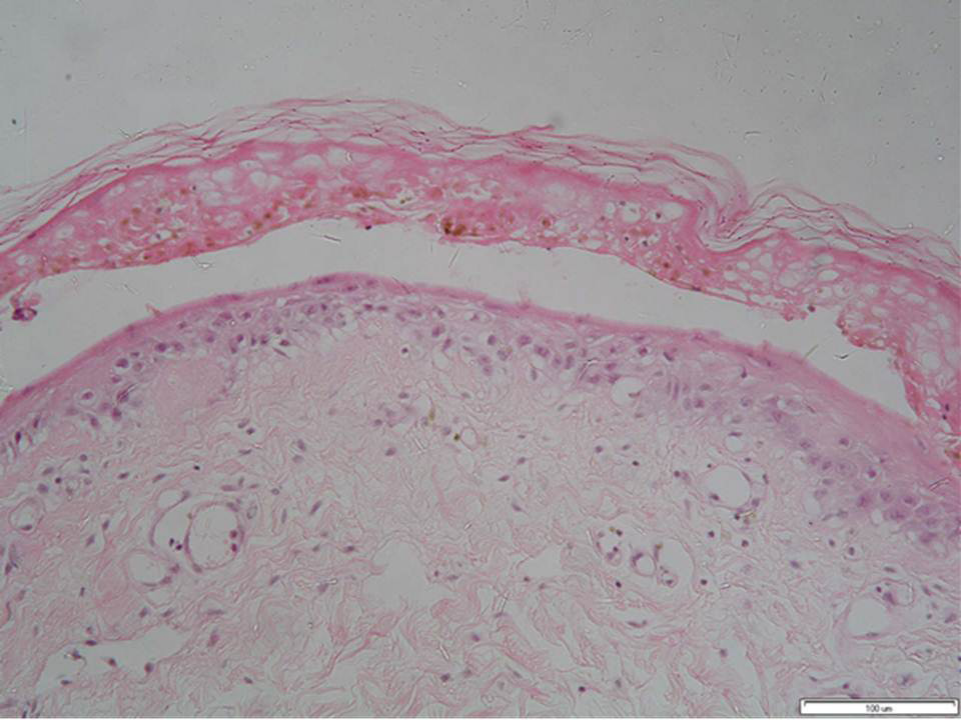
**A histopathological specimen (stained with hematoxylin and eosin) showing typical features of toxic epidermal necrolysis: acute interface dermatitis, extensive full thickness keratinocyte necrosis and vascular degeneration of the dermoepidermal junction (×200)**.

**Figure 3:**
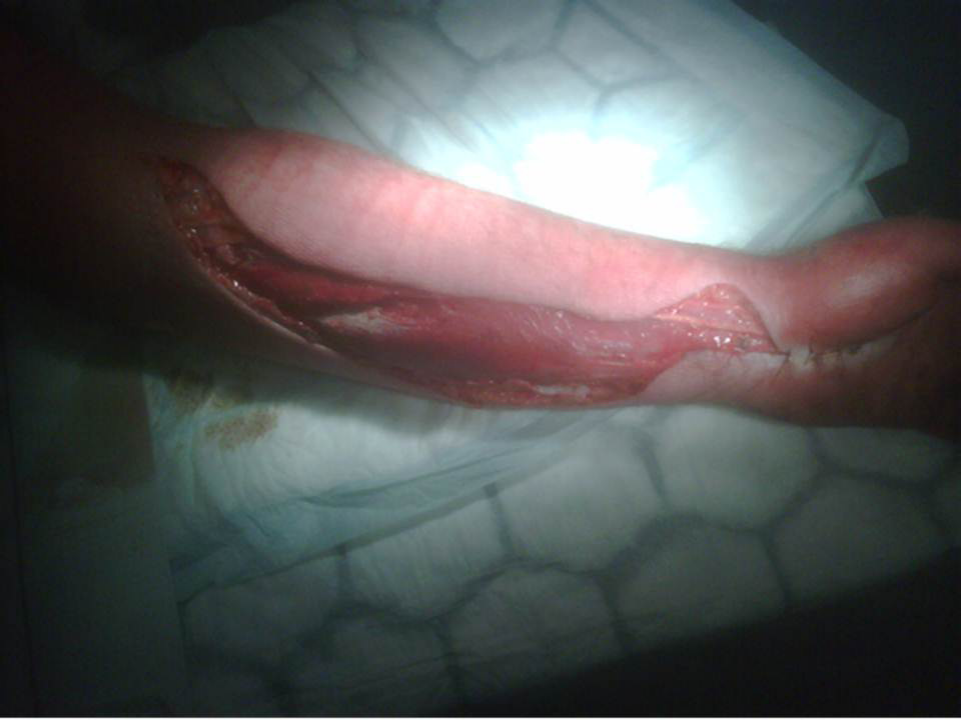
**Left forearm and wrist after fasciotomy and median nerve decompression**.

We report a rare case of compartment syndrome in the forearm of a 62-year-old Caucasian man of northern European origin who was recently diagnosed as having gout and prescribed allopurinol at a therapeutic dose by his general practitioner. Within less than 12 hours of drug administration our patient noticed oral mucosal blistering and erosions, followed by the appearance of a vesicular rash on his back. The lesions rapidly progressed within 24 to 36 hours to involve nearly his entire body surface area affecting his face, neck, torso, both arms, legs and perineum (more than 90% of his body surface area was affected) with the development of atypical and confluent flat target lesions with bullae in their centers (Figure 1). The development of those lesions was followed by extensive cutaneous sloughing and was associated with conjunctivitis, high-grade fever and malaise. According to the consensus definition proposed by Bastuji-Garin *et al*. in 1993 [10], our patient was diagnosed as having toxic epidermal necrolysis with spots (detachment above 30% of the body surface area plus widespread purpuric macules or flat atypical targets). He was also evaluated according to the severity of illness score of toxic epidermal necrolysis (SCORTEN) [11] and given a score of 5 (he was over 40 years of age, with no malignancy, more than 10% of his body surface area detached, he had tachycardia, elevated serum urea and serum bicarbonate, and normal glucose levels). He was admitted to the hospital under the care of a medical team for investigation. Full blood count, blood glucose, serum urea, creatinine, bicarbonate and electrolyte tests and oral, rectal and skin biopsies were taken (Figure 2) and a diagnosis of TEN with spots was made following dermatological review with acute renal and hepatic function impairment. He was started on supportive intravenous antibiotics, immunoglobulins (IGg), fluid resuscitation and allopurinol administration was stopped. The orthopedic team was asked to examine him later that day following his experiencing excruciating pain in his left forearm and hand (dominant arm) that was not responding to analgesia. On suspicion of compartment syndrome, compartment pressures were measured in both limbs on the ward. Compartment pressures were found to be elevated in both anterior (75 mmHg) and posterior compartments and deep (65 mmHg) in the left forearm. He was taken to the operating room and immediate fasciotomies were carried out with decompression of the median nerve in his carpal tunnel. His symptoms improved post-operatively and with the improvement in his renal and skin conditions he was taken to the operating room within 48 hours for wound inspection and successful secondary closure (Figure 3). He was admitted to the intensive care unit for two weeks and then discharged to the general medical ward for rehabilitation with a total duration of five weeks of hospitalization. Following this, he had a full recovery and was discharged with no residual musculoskeletal damage.

## Discussion

TEN syndrome is an uncommon, acute, life-threatening, medication-induced disorder with a reported mortality rate of up to 60% [8]. Different variables have been identified as risk factors. Drug-induced TEN is the main documented cause of TEN. More than 100 different drugs are considered as having caused TEN, but only a minority of them accounts for the majority of cases. These principal culprits are anti-infectives such as cotrimoxazole and sulfonamides, allopurinol, carbamazepine, phenytoin, phenobarbital, NSAIDs of the oxicam type, acetic acid NSAIDs, macrolides, quinolones, cephalosporins and tetracyclines [5]. The management of such patients is primarily supportive, with particular attention to body fluid balance, nutritional status, pain relief, early treatment of eventual sepsis, and meticulous skin care [3]. Recently, high-dose intravenous immunoglobulins have been advocated to treat TEN in combination with optimal care in a burn unit [9], as sepsis is a serious complication in patients with TEN [3].

Acute compartment syndrome (ACS) is defined as the development of increased pressure in a confined anatomic space that compromises circulation and leads to ischemia of the contained muscles and nerves. Several theories have been proposed regarding the pathophysiology of ACS. The arteriovenous gradient theory is arguably the most popular. It states that increased tissue pressure increases local venous pressure. This increased venous pressure approaches the pressure in the supplying arteries thus decreasing the arteriovenous pressure gradient. Local blood flow decreases as the gradient decreases and oxygen delivery to the surrounding tissue drops below demand. Muscle ischemia results in myocyte lysis and the release of toxic intracellular chemicals into the extracellular space. Microvascular damage leads to capillary leak, edema, and an increased compartment pressure. Muscle ischemia eventually progresses to muscle death. Over time the muscle is replaced with dense fibrous tissue, which results in limb contracture. Ischemic contracture, first described by Volkmann in 1881, is one known endpoint of untreated increased pressure within a confined forearm compartment. This pathologic event results in an atrophic and severely contracted extremity with muscle and nerve fibrosis.

Immunopathological mechanisms of TEN include recruitment of cluster of differentiation 4 (CD4)+ T cells in the dermis and CD8+ T cells in the epidermis thus inducing a T helper (TH)1 cell reaction [3]. Recruited memory CD4+ and cytotoxic CD8+ T cells produce interferon γ (IFNγ) and tumor necrosis factor α (TNFα), both triggering massive apoptosis of keratinocytes through one of two main mechanisms: the perforin/granzyme B mechanism and lytically active Fas ligand (Fas-L) mechanism [12]. Moreover, the production of TNFα would increase the expression of major histocompatibility complex (MHC) class I antigens in keratinocytes, making them more sensitive to cytotoxic cells producing perforin and granzyme B [3]. Recently in 2008, Chung *et al. *demonstrated that the cytolytic protein granulysin is probably the most important factor in epidermal apoptosis observed in TEN. Its concentration in the blister fluid was several orders of magnitude higher than that of other cytokines, such as perforin, granzyme B or Fas-L and this concentration was found to be positively correlated with disease severity [13]. Later, functionally active cells carrying the killer effector receptor CD94/NKG2C were detected in the blister fluid and the peripheral blood of patients with TEN and the authors postulated that this receptor might be involved in triggering cytotoxic T cells in the acute stage of the disease [14]. Some studies have shown that apart from acting as target cells in TEN, keratinocytes may also be cytolytically active through their further production of TNFα and Fas-L and hence trigger even more massive apoptosis [3].

On occasion, for reasons that are not entirely clear, the inflammatory response may spiral out of control, progressing until it involves the entire body. The circulatory system loses its integrity followed by the breakdown of the adherens junctions between the endothelial cells lining the capillaries, allowing fluid to leak into the tissue spaces. Trauma from electrical injuries and burns, and snakebite or envenomation injuries may cause a cascade of events that lead to the rapid formation of edema. Similarly, thermal and chemical burns may incite a cascade of events involving substantial tissue injury and systemic response with associated increases in vascular permeability and interstitial edema. It is important to recognize that compartment syndrome has been described in the uninjured limb of up to 2% of patients with burns, occurring as the result of a systemic inflammatory response and the requirement for extensive fluid resuscitation.

We believe that in the case of our patient, the combination of capillary endothelial damage with subsequent increase of capillary permeability and interstitial fluid edema in addition to the inflammatory response affecting the dermis associated with a certain degree of loss of skin elasticity predisposed our patient to compartment syndrome. Similar conditions have been noted in patients with burns and have suggested early limb escharotomies or fasciotomies in patients with full-thickness burns with close monitoring of peripheral circulation [15]. To the best of our knowledge there have been no documented similar cases (investigated via PubMed searches or any associated references in dermatology textbooks (Rooks)).

## Conclusions

TEN, despite its rare incidence, is a condition with a high incidence of complications and mortality. Patients with severe conditions with a large degree of affected surface area should be treated as promptly and effectively as patients with burns, with close monitoring and anticipation that rare musculoskeletal complications might arise. The association of compartment syndrome and TEN might lead to a rapid deterioration and fatal systemic involvement and multiple organ failures.

## Consent

Written informed consent was obtained from the patient for publication of this case report and any accompanying images. A copy of the written consent is available for review by the Editor-in-Chief of this journal.

## Competing interests

The authors declare that they have no competing interests.

## Authors' contributions

TK analyzed and interpreted the data from our patient, made the initial diagnosis and started treatment and was a major contributor in writing the manuscript. SE helped in analysis and patient management and was a major contributor in writing the manuscript. HM analyzed and interpreted the data from our patient regarding dermatological disease, performed the histological examination of the skin and mucous membranes and was a major contributor in writing the manuscript. JC was the supervising consultant and analyzed and interpreted the data from our patient, made the initial diagnosis and started treatment. All authors read and approved the final manuscript.
